# Advancements in Free-Radical Pathologies and an Important Treatment Solution with a Free-Radical Inhibitor

**Published:** 2018-02-21

**Authors:** RC Petersen, MS Reddy, P-R Liu

**Affiliations:** 1 Departments of Biomaterials and Restorative Sciences, University of Alabama at Birmingham, USA; 2 Office of the Dean, School of Dentistry, University of Alabama at Birmingham, USA; 3 Department of Restorative Sciences, University of Alabama at Birmingham, USA

**Keywords:** Free radical, Molecular oxygen, Reactive oxygen species, Reactive secondary sequence, Polymerization, Lipid peroxidation, Membrane fluidity, Free-radical inhibitor

## Abstract

Unsaturated carbon-carbon double bonds particularly at exposed end groups of nonsolid fluids are susceptible to free-radical covalent bonding on one carbon atom creating a new free radical on the opposite carbon atom. Subsequent reactive secondary sequence free-radical polymerization can then continue across extensive carbon-carbon double bonds to form progressively larger molecules with ever-increasing viscosity and eventually produce solids. In a fluid solution when carbon-carbon double bonds are replaced by carbon-carbon single bonds to decrease fluidity, increasing molecular organization can interfere with molecular oxygen (O_2_) diffusion. During normal eukaryote cellular energy synthesis O_2_ is required by mitochondria to combine with electrons from the electron transport chain and hydrogen cations from the proton gradient to form water. When O_2_ is absent during periods of irregular hypoxia in mitochondrial energy synthesis, the generation of excess electrons can develop free radicals or excess protons can produce acid. Free radicals formed by limited O_2_ can damage lipids and proteins and greatly increase molecular sizes in growing vicious cycles to reduce oxygen availability even more for mitochondria during energy synthesis. Further, at adequate free-radical concentrations a reactive crosslinking unsaturated aldehyde lipid breakdown product can significantly support free-radical polymerization of lipid oils into rubbery gel-like solids and eventually even produce a crystalline lipid peroxidation with the double bond of O_2_. Most importantly, free-radical inhibitor hydroquinone intended for medical treatments in much pathology such as cancer, atherosclerosis, diabetes, infection/inflammation and also ageing has proven extremely effective in sequestering free radicals to prevent chain-growth reactive secondary sequence polymerization.

## Introduction

Free-radical polymerization is one of the most important chemistries in the world today key to many diverse applications including use in the development for various types of aerotech structures, aircraft, marine manufacturing, commercial/military cars and trucks, ballistic material, medical bone cements, many dental restoratives, water resistant surface protection and repairs. A breakthrough in free-radical chemistry is distinguished by reactive secondary sequence covalent bonding through multiple carbon-carbon double bonds especially during intermittent hypoxia that provides significant understanding to most known medical states [1–3]. Free radicals are extremely unstable molecules with an unpaired electron in an outer valence orbital that needs an extra electron to restore stability [4–6]. Unsaturated carbon-carbon double bonds particularly at exposed end groups are especially susceptible to a free-radical forming a covalent single bond on one carbon atom to create a new free radical on the opposite carbon atom [1,2]. Reactive secondary sequence free-radical bonding can then continue a polymerization chain-growth reaction across numerous carbon-carbon double bonds to form larger molecules or ultimately macromolecular polymers [1,2].

Cellular mitochondrial organelles produce over 90% of the adenosine triphosphate for the cell during aerobic energy synthesis [7,8]. As a result, mitochondria consume approximately 85% of all cellular O_2_ [9]. However, during hypoxic aerobic conditions mitochondria also produce electrons that are the chief source for free radicals as reactive oxygen species (ROS) like superoxide anion (O_2_^•−^) by the one electron reduction of O_2_ [9–16]. ROS comprise O_2_^•−^, hydrogen peroxide (H_2_O_2_) and the hydroxyl radical (HO^•^) [1,2,9,17]. The free radical O_2_^•−^ and HO^•^ are unstable molecules with an unpaired electron [1,2]. Alternatively, H_2_O_2_ is a moderately stable molecule but can produce HO^•^ when exposed to transition metal cations for example divalent ferrous iron or Fe^+2^ [1] common to the heme molecule [18] and found in connective tissue [19,20]. High levels of ROS generated through mitochondria can cause damage to lipids, proteins and DNA [17,21–30]. Further, ROS can augment pathology [1,3,15,17,22,25,27,30] and even increase ageing [9,19,20,24,31]. On the other hand, ROS can provide a level of biology for physiologic protection at low concentrations [17,21,22,30,32–36].

**Figure 1:**
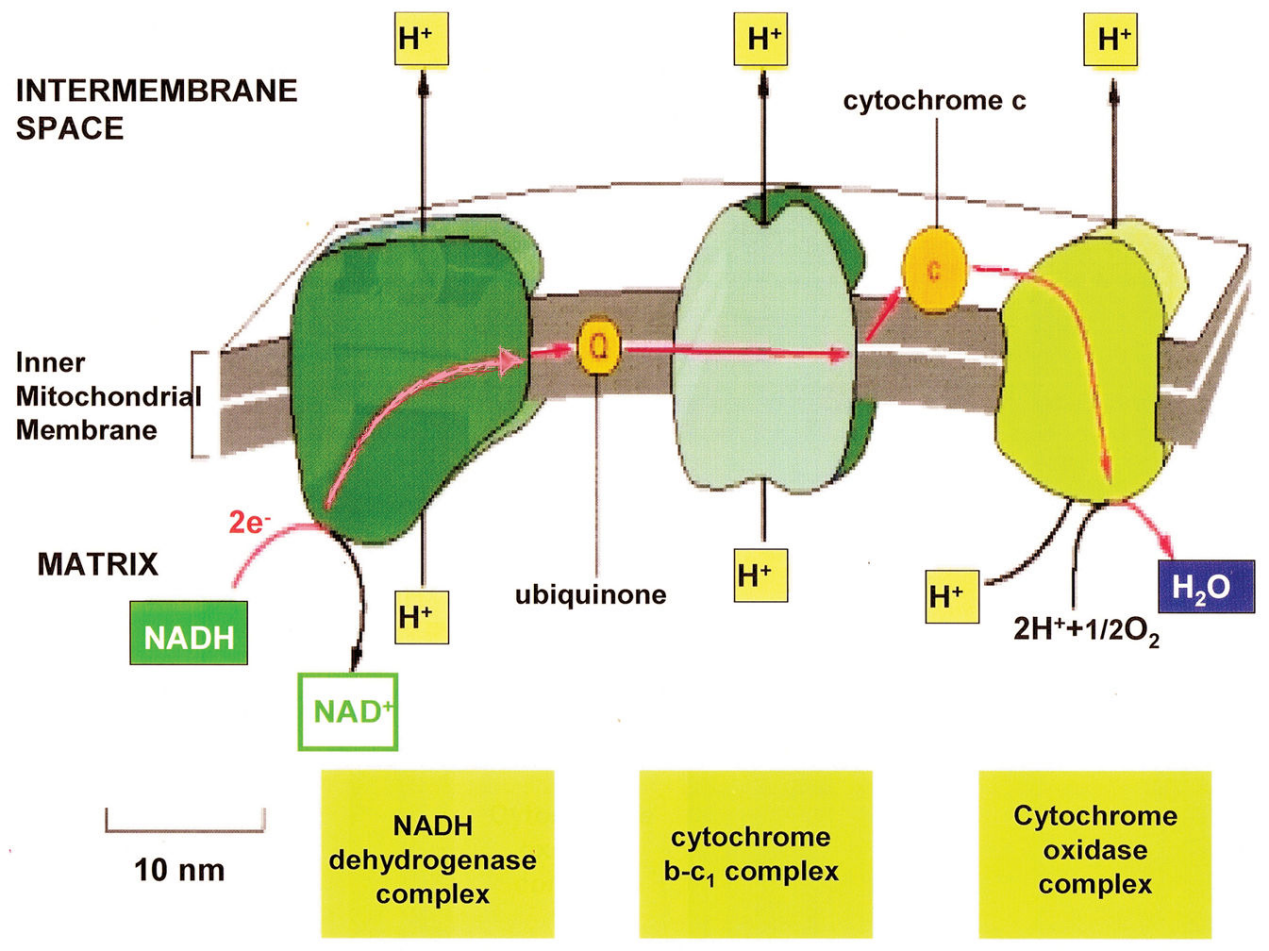
O_2_ is needed at the end of the electron transport chain in removing electrons and protons to form H_2_O. (Molecular Biology of the Cell. 4^th^ edition. Electron-Transport Chains and Their Proton Pumps. Figure 14–26. Copyright © 2002, Bruce Alberts, Alexander Johnson, Julian Lewis, Martin Raff, Keith Roberts, and Peter Walter; Available from: http://www.ncbi.nlm.nih.gov/books/NBK26904/).

Mitochondria require O_2_ during energy metabolism so that electrons generated through the electron transport chain and protons that develop around the proton gradient can combine with help from proteins that are enzymes to form water (Figure 1). In the process of mitochondrial aerobic metabolism NADH is oxidized to NAD^+^ releasing 2 electrons and a proton. Without O_2_ during mitochondrial energy synthesis excess electrons can form free radicals and protons can produce acid [1,2]. When combined with acid free radicals can break down large lipids such as polyunsaturated fatty acids (PUFAs) and with possible help from enzymes produce smaller reactive unsaturated aldehydes that greatly increase free-radical crosslinking reactive secondary sequence polymer chemistry [1,2]. Lipids are classified by two types as simple fats and waxes with hydrolyzable ester linkages between fatty acids and glycerol or as complex ring structures like cholesterol and other steroids without ester linkages [37]. Simple lipids are further categorized by the fatty acid chains as fats or oils in relation to the amount of bond saturation or also carbon-carbon double bonds respectively [37,38]. When the number of carbon-carbon double bonds is lowered saturation is increased with greater amounts of hydrogen-carbon bonds for higher melting points and unsaturated low viscosity oils can become solid-like saturated fats [37]. Free radicals produced as a result of O_2_ structural barriers and decreased O_2_ diffusion to mitochondria are then possibly accessible for chain growth polymerization crosslinking with the reactive unsaturated aldehydes by covalent bonding across PUFA double bonds to increase molecular structure to a great extent [1,2]. Subsequent PUFA double-bond reactive secondary sequence crosslinking can then generate an increasing run-away spiral of molecular barrier structures to O_2_ diffusion and lower O_2_ availability for mitochondria during energy synthesis resulting in continuing ever-greater production of free radicals [1,2].

**Figure 2:**
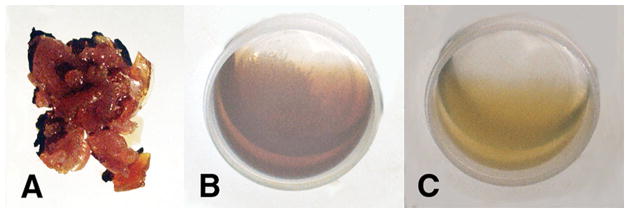
(A) Unsaturated fatty acid lipid oils, benzoyl peroxide free-radical initiator, cobalt naphthenate transition metal accelerator and α,β-unsaturated aldehyde reactive lipid breakdown product acrolein crosslinker polymerized into solid rubbery gel. (B) Unsaturated fatty acid oils, benzoyl peroxide and cobalt naphthenate accelerator remain unreacted low-viscosity oil without acrolein crosslinker. (C) Unsaturated fatty acid lipid oils, benzoyl peroxide, and acrolein α-β unsaturated aldehyde remain unreacted low-viscosity oil without cobalt metal free-radical accelerator. (Micromechanics/Electron Interactions for Advanced Biomedical Research, 2011, Chapter 16. Free Radical Reactive Secondary Sequence Lipid Chain-Lengthening Pathologies. Figure 10. Richard Petersen and Uday Vaidya).

**Figure 3:**
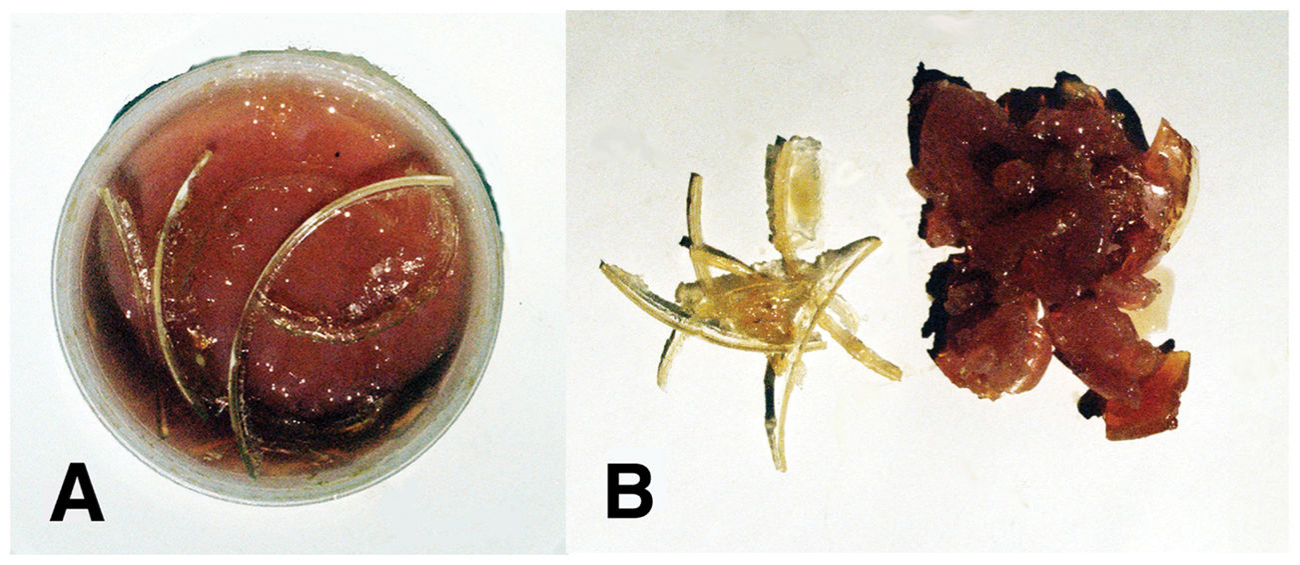
Differences between free-radical polymerized reaction products for lipid peroxidation across oxygen-oxygen double bonds and unsaturated lipid reactive secondary sequence polymerization along carbon-carbon double bonds. (A) Reactive secondary sequence free-radical polymerization with crosslinker and unsaturated lipids form solid rubbery gel on the bottom. Crystalline polymerization lipid peroxidation products were pulled off the sides of the reaction container that concentrated alongside of the nonpolar polyethylene container surface. (B) Left Side-crystalline lipid peroxidation polymerization products of acrolein crosslinked lipids and O_2_ and Right Side-reactive secondary sequence polymerized unsaturated lipids in a solid rubbery gel phase. (Micromechanics/Electron Interactions for Advanced Biomedical Research, 2011, Chapter 16. Free Radical Reactive Secondary Sequence Lipid Chain-Lengthening Pathologies. Figure 12. Richard Petersen and Uday Vaidya).

Free radicals can interact for increased structural organization by molecular crosslinking to reduce O_2_ transport at the molecular, cellular, tissue and vascular levels that may generate pathology in cancer, atherosclerosis, diabetes, trauma, inflammation and infection as basic examples. Free-radical ROS at the normal low levels are considered to be part of cell physiology for example with antimicrobial oxidative bursts to destroy microbes, control autophagy to reuse intracellular organelles or molecules by a type of nutrition biosynthesis, provide a form of cell signaling as a way of adapting to stress and following trauma support healing in association with molecular growth factors [17,21,22,30,32–36]. But, at higher free-radical levels molecules such as lipids, proteins and DNA can be damaged [17,21–30]. When free-radical crosslinking occurs between unsaturated lipids especially in the presence of low molecular-weight reactive unsaturated lipid aldehyde breakdown products the low oil viscosity can increase and eventually produce rubbery solids and even crosslink between the double bonds of O_2_ concentrated near a nonpolar interface to generate crystalline lipid peroxidation products [1] (Figures 2 and 3). With sufficient concentration levels of free-radicals and reactive unsaturated aldehydes the unsaturated lipid polymerization process is thermodynamically favorable even at just room temperature [1]. During covalent free-radical reactive secondary sequence crosslinking lipid oils increase viscosity [1,2] and cell membranes can draw together to become less rounded with decreased fluidity so that molecular oxygen diffusion through the membrane is reduced [2]. Also, free radicals can stiffen the extracellular matrix as in many cancers [39–45]. Further, by free-radical reactive secondary sequence polymerization the lipid core of plaque lesions in atherosclerosis can develop hardness with size enlargement [1,36] as part of a complex process that may restrict blood flow [36]. Multiple initiating events that start the cascading reductions in transport of O_2_ could include many combined factors from toxicity of environmental chemical interactions or improper nutrition, ionizing radiation, tissue trauma with irregular healing and scarring, various infections coordinated with damaging inflammation, vascular obstructions, increased extracellular matrix stiffness, or reduced cell membrane fluidity to name a few associated events or initiating sources.

## Cell Membranes

Free radicals can result in reducing membrane fluidity to increase membrane rigidity [2,9,46–50]. Most importantly, free radicals target PUFAs that lower in content as a sign crosslinking occurs with loss of carbon-carbon double bonds [46–50]. When the fatty acid saturated/unsaturated ratio increases membrane fluidity can also decrease due to increased molecular packing by saturated single bond rotation entanglements compared to unsaturation with carbon-carbon double bonds that reduce bond entanglements [2,51,52]. Further, membrane fluidity can decrease by increasing the fatty acid chain lengths [51]. Conversely, membrane fluidity can increase during free-radical electrophilic attack with hydrolysis on PUFA carbon-carbon double bonds [2,53,54] by oxidative cleavage forming smaller molecules as relatively unreactive aldehydes that can travel easier into less significant spaces [1]. Increased fluidity occurs as a result of smaller molecules increasing diffusion exponentially whereas longer molecules decrease movement by single bond rotation entanglements [55]. The Fluid Mosaic Membrane Model offers the best accepted insight into limitations on lateral diffusion of lipids and proteins within the membrane [56,57]. The present Fluid Mosaic Membrane Model illustrates how lateral protein mobility is dependent on membrane fluidity along with protein size or protein aggregation [58]. As a result, within the membrane lateral molecular movement allows lipids and proteins to search for molecules of similar covalent polarities by weak attractive forces [2]. Saturated lipids that form as more rigid-like macromolecules from unsaturated lipid oils to reduce more fluid membrane regions appear to be influenced through free-radical ROS crosslinking into lipid rafts by demonstrating increased saturated amounts with increasing H_2_O_2_ [50]. Structural rigidity organization of the plasma cell membrane increases for lower fluidity as a consequence of ageing [9]. Further, oxidative damage decreases fluidity of the inner mitochondrial membrane [9]. Plasma cell membrane unsaturated fatty acids and especially PUFAs are especially at risk to ROS electrophilic attack [49,50] because of susceptible carbon-carbon double π bonds that in turn create lipid products with saturated single σ bonds [1,2]. Regarding other ROS influences to increase membrane rigidity and reduce molecular lateral diffusion, proteins are found to agglomerate or crosslink when exposed to ROS for example by dityrosine crosslinks with metal catalyzed reactions [59–61], through cysteine disulfide crosslinks [62,63] or from the reactive unsaturated aldehyde acrolein that crosslinks amino acids with serine, histidine, arginine, theronine and lysine being most susceptible [64].

Erythrocytes with high concentrations of PUFAs exposed to ROS experience reduced fluidity that indicates free radicals are involved by crosslinking [48,49]. Also, erythrocyte membranes deform when exposed to ROS developing a pointed extension [49] similar to pointed membrane extensions that are traits of free-radical crosslinked cancer cells [3]. Consequently, PUFA free-radical crosslinked stiffer membranes during irregular hypoxic conditions with mitochondrial energy synthesis could explain loss of membrane fluidity and resultant pathology [2]. In addition, longevity is thought to increase with lower fatty acid unsaturation levels due to less exposure of carbon-carbon π bonds vulnerable to ROS attack [65].

Nonpolar O_2_ diffuses through the nonpolar cell membrane phospholipids by similar polarity attractions to ultimately combine with excess electrons and protons during mitochondrial energy synthesis [1,2]. Also, lateral motion of lipids and proteins in the membrane requires sufficient fluidity [58]. But, with irregular hypoxic conditions electron radicals can generate from the mitochondrial electron transport chain and hydrogen cations can increase from the proton gradient [1,2]. As a result of hypoxic environments, membrane fluidity can be decreased by free-radical crosslinking with PUFAs [9,46–50]. ROS also influence lowering membrane fluidity and reduced molecular lateral diffusion by agglomerating or crosslinking proteins [59–64]. Resultant reductions in membrane fluidity consequently interfere with diffusion of O_2_ [66–68]. Less O_2_ available for normal mitochondrial energy synthesis in turn produces more free radicals [1–3]. Consequently, an escalation of free-radical concentrations over time can increase membrane PUFA crosslinking to reduce membrane fluidity with increased macromolecular barriers to decrease O_2_ diffusion even more [2]. As free-radical concentrations rise molecular degenerative insults accumulate on lipids, proteins and DNA [17,21–30]. Subsequent build up of free-radical damage is considered an important component for many medical conditions [1,3,17,22,25,27,30] and even ageing [9,19,20,24,31].

## Cancer

**Figure 4:**
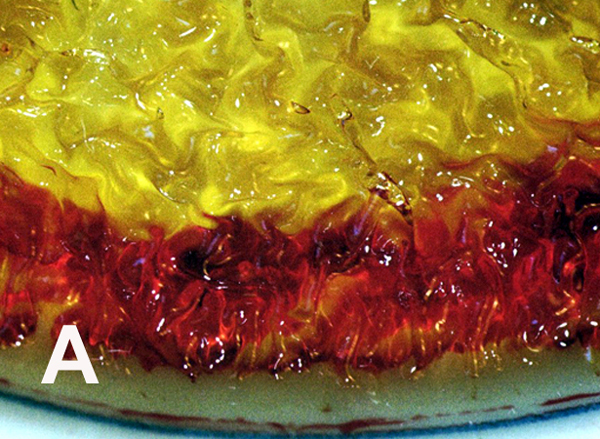

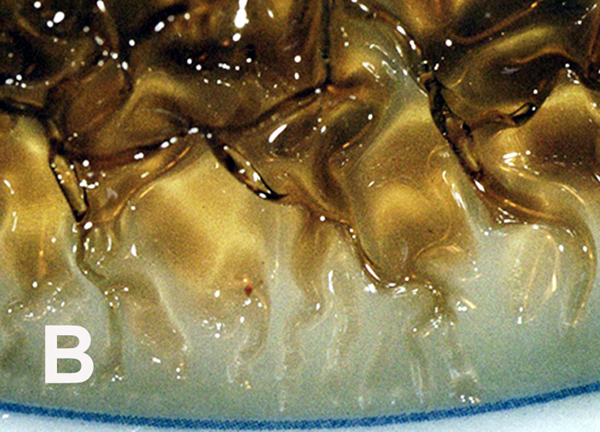
Free-radical polymerization of vitamin supplements containing numerous multiple unsaturated carbon-carbon double bonds and without acrolein crosslinker generates rubbery solid gels from low viscosity oils. (A) β,β-carotene. (B). Vitamin A (Micromechanics/Electron Interactions for Advanced Biomedical Research, 2011, Chapter 16. Free Radical Reactive Secondary Sequence Lipid Chain-Lengthening Pathologies. Figure 16. Richard Petersen and Uday Vaidya).

**Figure 5:**
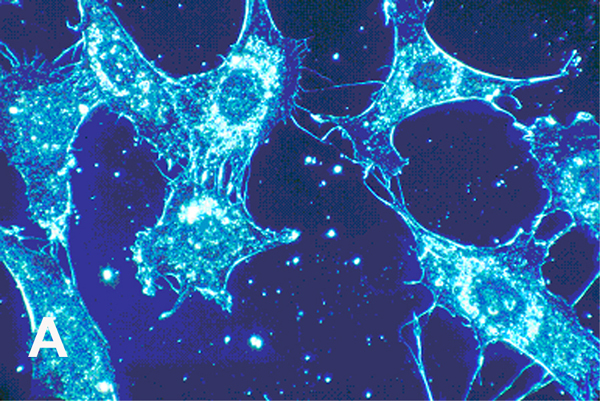

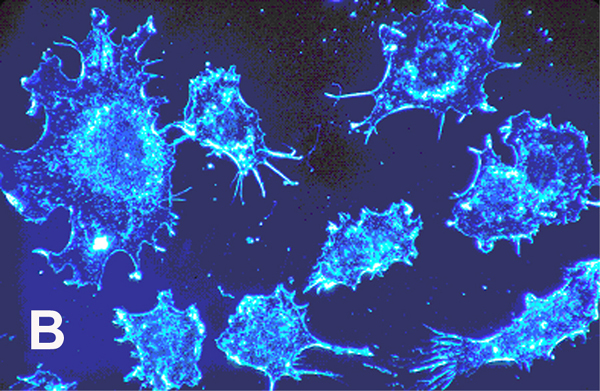
Cell cultures from human connective tissue 500× (A) Normal cells with smoother membrane borders. (B) Cancer cells with more spike-like protrusions revealing more irregular deeper plasma cell membrane invaginations. (With permission from the National Institutes of Health/Department of Health and Human Services).

As presented previously, elevated pathologic ROS levels and oxidative damage can decrease membrane fluidity [2,9,46–50]. Hypoxia in cancer cells produces high concentrations of free radicals [3,69–77], expected by irregular O_2_ availability to mitochondria during energy synthesis to generate the superoxide anion O_2_^•−^ [9,15,16,31,54,78–80]. Cancer cell membranes reflect oxidative stress by ROS with uneven distorted borders, membrane ruffling and irregularly shaped nuclei compared to smoother more even rounder membranes of normal cells with smooth nuclei [3]. A notable expression of free-radical covalent bond polymerization is the linear or volumetric shrinkage from the original fluid-like material toward a harder and smaller more solid mass [1–3,81–84]. Also, free-radical polymerization shrinkage creates a distortion or warpage at some level because of uneven covalent bonding [1–3,81,83,84]. Uneven nonuniform polymerization shrinkage with warpage is enhanced with film coatings having uneven depth without smooth regular support underneath [84]. Cytoskeletal actin fibers provide strength inside the plasma cell membrane [85]. Unsaturated lipid oils of the plasma cell membrane would then crosslink over supporting actin fibers set irregularly beneath to increase polymerization shrinkage warpage [3]. Unsaturated lipids pulled together by free-radical covalent crosslinking at rounder more even membrane borders would need invagination to wrinkle inward particularly when coupled with irregular actin fiber support underneath to explain the distorted appearance that occurs during the transformation to cancer cells [3]. Related to irregular uneven cancer cell membranes, vitamin A and β,β-carotene nutrient capsules both with numerous multiple carbon-carbon double bonds in unsaturated oil suspensions produced extensive wrinkling and warpage during free-radical polymerization into solid rubbery gels [1] (Figure 4). Similar to carbon-carbon double bond polymerization shrinkage with warpage, cell cultures demonstrate comparisons between normal smoother membranes in contrast to cancer cells with more distorted uneven membranes that include spike-type extensions to create deeper invaginated borders (Figure 5).

**Figure 6:**
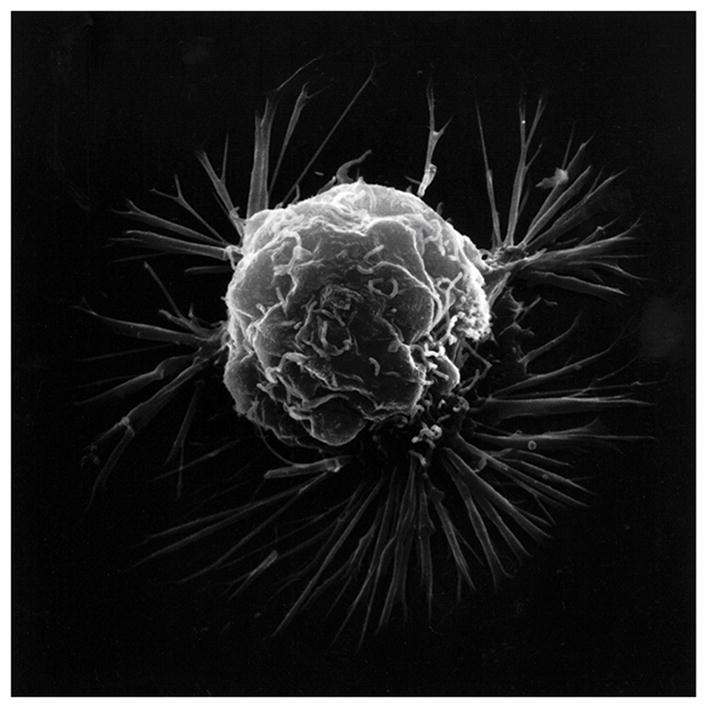
Scanning electron microscopy of an isolated cancer cell with membrane ruffling and long lamellipodia spike-like extensions. (With permission from the National Institutes of Health/Department of Health and Human Services).

Cancer transformation entails cell movement through an epithelial-mesenchymal transition (EMT) with alterations in cell shape and invasion of neighboring tissue [85,86]. Cancer cells have shown motility responses to ROS with H_2_O_2_ that can degrade into HO^•^ and produce projections at the cell membrane borders [87,88]. Chemotaxis has been sustained by ROS as H_2_O_2_ to direct chemotaxis by controlling chemoattractants that connect to the cell membrane with actin polymerization for cell movement toward H_2_O_2_ and other ROS [89–91]. Cell membrane projections can generate adhesive attachments to the extracellular matrix that are able to contract as molecular bonds develop to advance the cell forward [87,92,93]. Mitochondrial electrons appear capable of delocalizing during oxidative stress through microtubules and actin fibers out to the plasma cell membrane [3]. Free-radical covalent bond crosslinking and weak secondary bonding supply a method for contraction that can bring large macromolecular structures closer together [1,82–84] and provide forward cell movement [2,3]. Polymerization of actin can extend fibers outward from the plasma cell membrane creating projections that contain focal adhesions to the extracellular matrix and advance the cell forward as adhesive bonds form to contract [87,92,93]. Cancer plasma cell membranes demonstrate the irregular membrane borders with ruffling and wide spike-like projections lengthening away from the cell (Figure 6).

**Figure 7:**
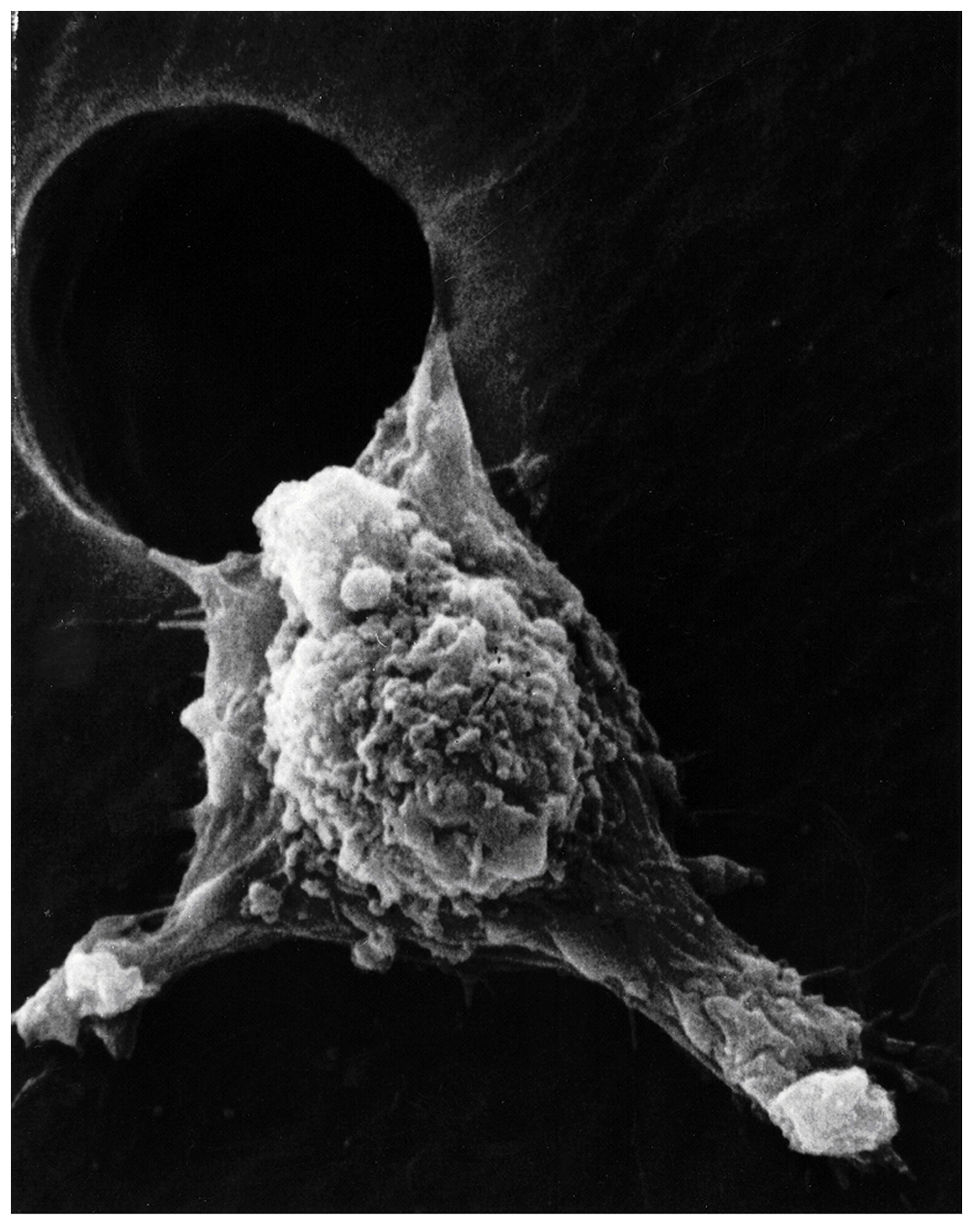
Metastasis scanning electron micrograph of a low modulus cancer cell moving through an artificial hole showing stiff pseudopodia extensions called lamellipodia. (With permission from the National Institutes of Health/Department of Health and Human Services).

Despite spike-like projections extending from the cancer plasma cell membrane that could interfere with mobility, invasive cancer cells have smaller membrane surface areas with lower modulus to provide flexibility and better facilitate squeezing through small spaces such as openings in the blood vessel endothelium [94]. Nevertheless, spike-like projections are a strong characteristic on the leading edge of cancer cells during metastatic movement [95]. Actin fibers align along the axis of the membrane extensions for highest modulus to resist sideways deflections in the forward direction [95]. As the stiff membrane extensions squeeze through small spaces leverage can be applied to force such narrow openings further apart to help invade new tissue [95] (Figure 7). Fibers of the cytoskeleton conduct electrons from the negative centrosome near the nucleus to the positively charged outer plasma cell membrane surface side as radical negatively charged electrons to provide polymerization chemistry for advancing actin fibers [3]. Electrons conducted through microtubules to actin fibers [96] are generated in excess by mitochondria under irregular oxidative conditions with hypoxia [3]. Actin has shown reorganization during exposure to free radicals from H_2_O_2_ that increase cell movement [97]. In terms of free-radical initiation, H_2_O_2_ has been shown to be excellent for polymerizing polyester resins [98]. By analogous ROS free-radical chemistry, oxidized low density lipids have demonstrated the ability to produce actin polymerization in macrophages [99]. Of great concern, H_2_O_2_ with other ROS are found in many cancer cells [3,69–77,100].

Metastatic cancer cells have a lower modulus (or less stiffness approximately) to deform more easily than normal cells and also show pleomorphic smaller sizes with less membrane area [101–103]. Cell modulus increases with actin fiber cytoskeleton organization, but in cancer actin fibers become disordered where cells become less stiff and more easily distort [103]. Conversely, tumor tissue density increases as a risk factor in cancer [40,104–106]. Higher tissue density reflects greater amounts of extracellular matrix collagen [40,104], collagen crosslinking [42,45], and increasing stiffness [42,45] to provide increased traction for cancer cell focal adhesions that improve cell motility [106,107]. Stiffer tumor substrata found in higher density fibrotic areas with increased collagen promote ROS at the cancer cell membrane to activate the EMT for cancer cells during tumor metastasis [43]. Also, cells tend to move toward stiffer substrates of higher modulus [107]. Stiffer collagen tissue would interfere with O_2_ diffusion to account for promoting ROS from associated mitochondria nearby the hypoxia of the plasma membrane. Further, ROS response near the plasma membrane in contact with increased fibrotic collagen should include creation of focal adhesions at the leading edges to contract by free-radical covalent bonding that pull the cell forward toward the stiffer tumor tissue and better explain EMT cancer cell motility. By possible relationship to ROS and EMT cancer cell movements, lysyl oxidase is an enzyme that promotes extracellular matrix collagen crosslinking and stiffness [108–110] as a hypoxia-related protein which is associated with cancer metastasis [41,111].

## Atherosclerosis

Atherosclerosis known by “hardening of the arteries” is a foremost medical problem that embodies extensive disease linked with high ischemic-related mortality [28,112–114]. Arterial stiffness appears to be associated with oxidative stress [115–119] and increased extracellular matrix collagen deposition crosslinking [120]. In addition to advancing arterial stiffening, atherosclerosis represents systemic pathology where lipids infiltrate into the vessel walls with inflammation, cells and fibrotic scar tissue to produce appreciable narrowing of the main susceptible arteries and the foundation for most cardiovascular disease [112–114,121]. Further, from National Institutes of Health records by gross pathology imaging, considerable lipid-rich solids can form to accumulate directly in a vessel lumen [1] that suggests intense free-radical covalent chemistry is involved. Ischemia can cause a heart attack with infarct or a stroke and brain damage when blood flow is interrupted to the heart or brain, respectfully [36,112,113]. Free radicals attack carbon-carbon double bonds in the alkene PUFAs that increase the risks for cardiovascular disease [122], while ROS generated by mitochondria are considered an important part of the development for atherosclerosis [28,36,121,123]. Further, alkanes form saturated solid fats by molecular single bond rotation entanglements whereas planar alkene carbon-carbon double bonds decrease molecular bond entanglements to form unsaturated oils [2,37,38,51,52]. Following chronic free-radical buildup with oxidative cleavage of PUFAs at lower pH to produce shorter reactive unsaturated lipid aldehyde crosslink products, reactive secondary sequence carbon-carbon double-bond chain-growth PUFA polymerization might include a loose interpenetrating network through the lipid core with other molecules like saturated lipids to alter fluidity of normal structures [1].

Extracellular lipid “fatty streak” deposition with inflammatory oxidized low density lipid-filled macrophage “foam cells” is the initial signal of atherosclerosis in coronaries for young adults and children [36,112–114]. Extracellular lipid pools form to accumulate that stain as esters amassed in both macrophages and the extracellular space [36]. Ester staining indicates the presence of simple lipids as triglyceride-fatty acids rather than complex lipid ring structured cholesterol [37]. During the development of atherosclerosis, endothelial oxidative stress is related to free-radicals and ROS [112,123–125], low density lipids [36,112,124,125], ischemia [125], inflammation [112,124,125], and infection [112,124,125]. Subsequent free radicals form through the mitochondria in periods of ischemia [9–16,112] that can support atherosclerosis [126,127]. Also, free radicals oxidize low density lipids that deposit in vessel walls [112,124], accumulate by neutrophiles during inflammation [128] and occur with infection [129–131]. Further, free radicals build up in all layers of the atherosclerotic wall [124]. The plaque central lipid core may also contain a crystalline lipid following necrosis of the macrophage foam cells [36]. By possible related chemistry, a crystalline lipid has been shown possible by free-radical unsaturated lipid crosslinking in connection with nonpolar O_2_ accumulation near a nonpolar interface that might be compared to a cell membrane surface [1]. Thick fibrotic lipid-rich-core plaques subsequently diminish O_2_ diffusion [36,112] that can speed up the production of mitochondrial free-radicals through ischemia [9–16,112]. Reduced O_2_ transport could produce both excess mitochondrial free radicals and acids that should be better acknowledged toward increasing the build up of lipid pathology at all stages and forms of disease [1]. When free radicals crosslink alkenes, subsequent oxygen diffusion is compromised even more to deeper inner layers [132–135].

Lipid peroxyl free-radical products due to crosslinking by O_2_ that can generate a hard lipid peroxidation crystalline-like material are a matter of more concern [1]. When harder lipid peroxidation crystal formation develops even at the molecular level, O_2_ diffusion to deeper layers would subsequently be even more restricted than with the rubbery gellation of reactive secondary sequence carbon-carbon double-bond crosslinking [1]. Saturation of a hydrocarbon polymer by removing carbon-carbon π double bonds and replacement with σ single bonds interferes greatly with electron travel and also changes more polar surfaces toward nonpolar [6,136] that needs some understanding for likeness between the weak intermolecular forces of attraction [137]. Nonpolar O_2_ concentration could exaggerate at a nonpolar insulating surface like nonpolar endothelial lipid cell membranes in an artery to create a possible nonpolar insulating free-radical sequestering condition that could develop with reactive lipid breakdown aldehyde acrolein crosslinking unsaturated lipids [1]. Increasing free radical concentrations then create the potential reaction circumstances for both combined double-bond molecular oxygen lipid peroxidation into crystal structure also with lipid alkene carbon-carbon double-bond reactive secondary sequence crosslinking into a solid lipid gel-like polymer [1].

## Diabetes

ROS are involved in the development of obesity or diabetes and further thought to promote insulin resistance [138]. High oxidative damaged lipids and proteins are found in different tissues of both type I and type II diabetes [138]. In type II diabetes mellitus the cell membrane PUFA: saturated fatty acid ratio reduces with an increase in membrane stiffness [52]. In addition to greater membrane rigidity by increased packing of saturated fatty acids with loss of unsaturated fatty acid oils [2,51,52], covalent crosslinks need consideration between carbon-carbon double bonds that pull molecules together at the molecular bond level [2]. Since type II diabetes mellitus is associated with ROS [22,139], the PUFA: saturated fatty acid ratio decrease with increased membrane stiffness [52] is also indicative of free-radical carbon-carbon double bond breakdown with an initial loss of unsaturated lipids and subsequent generation of lipid breakdown aldehydes that can produce percents of highly reactive unsaturated aldehyde products [1]. Lower molecular weight reactive unsaturated aldehydes then greatly promote free-radical carbon-carbon double bond reactive secondary sequence chain growth crosslinking with covalent structural rigidity [1]. Crosslinked unsaturated lipid chain growth organization structure could subsequently be apparent pathology chemistry for the increased membrane stiffness in type II diabetes mellitus [2]. An important concern regarding the free-radical lipid crosslinked membranes and lowered membrane fluidity would be to interfere with O_2_ diffusion [66–68] that could then increase associated diseases [2].

Reduced membrane fluidity can generate membrane interactions unfavorable for glucose transport with increased insulin resistance [52]. Insulin resistance develops through a complex manner in the cell membrane with multiple molecularly integrated network-type steps that interplay for insulin signaling [138]. Physiologic lower ROS concentrations with cell-signaling protein actions [30] might include molecular attractions favorable for insulin function. Conversely, clinically potential high ROS concentrations could combine at several levels to exacerbate diabetes and increase insulin resistance. As examples, lower membrane fluidity interferes with molecular diffusion, possible uneven crosslinked surface membrane irregularities with exposed lipid radicals might interfere with insulin membrane docking and the total of damaging protein modifications collectively could then severely limit insulin molecular performance at the outer plasma cell membrane.

Of particular pathologic alarm related to ROS associated with diabetes mellitus is the high risk of cardiovascular diseases including complications from renal damage, microangiopathy that causes blindness and heart attacks [139]. Increased membrane stiffness with reduced deformability caused by ROS in erythrocytes [48,49,52] and increased stiffness with plasma cell membranes of endothelial cells surrounding vessel walls [52] both of similar diameters could possibly reduce capillary blood flow to tissues resulting in hypoxia, nutritional deficiencies and microangiopathy [52]. Also, National Institutes of Health gross pathology imaging shows that lipid-rich solids can form directly in a vessel lumen [1] that may suggest concentrated free-radical crosslink chemistry to generate interference with blood flow at any level. Further, ROS associated diabetic microangiopathy extends to the bone marrow and may play a role in reducing hematopoietic stem cells that are needed for tissue repair and differentiation particularly in ageing individuals with diabetes mellitus and ischemic complexities [139]. Another severe complication of diabetes mellitus and secondary kidney damage that promotes inflammation with an increase in ROS is diabetic neuropathy [140,141].

## Infection and Inflammation Responses

Infection or tissue damage can elicit an inflammatory response [142–146]. During inflammation ROS are produced by the host cells to remove microorganisms [142–146]. Further, interruption of molecular oxygen at any level to mitochondria during energy synthesis can increase the production of electrons from the electron transport chain producing ROS and H_2_O_2_ [1–3,9–16,147]. Excess H_2_O_2_ that is not converted to water can travel through cell membranes and generate ROS as HO^•^ [147]. In the extracellular space ROS can produce damage to increase injury with increasing exaggerated immune responses [142–145,147]. Conversely, as tissue damage proceeds the inflammatory response may not control the infection to even promote invasive microorganisms deeper and expand initial micropathology into more severe clinical manifestations or chronic complications [142–145,147].

## Free-Radical Theory for Ageing Regarding Vitamins as Antioxidants and Clinical Error

The Free-Radical Theory of Ageing asserts that ageing is due to the accumulation of free-radical biologic damage which increases disease and death [19,20]. The chemical foundation was thought to be a result of free radicals produced by mitochondrial oxidative proteins that are enzymes through the energy synthesis process combined with the cation transition metal catalysts in the connective tissue [19,20]. After generation free radicals reacted within cells and tissues to begin the progression of ageing [19,20]. As one example, the accumulation of oxidative damage is thought to play a significant role in creating mitochondrial degeneration during ageing [9]. Consequently, the Free Radical Theory of Ageing indicated that treatment could begin with antioxidants to safeguard cells and tissues from free radicals to reduce ageing, increase lifetime and prevent disease [19,20]. Epidemiological research suggested that nutrition especially by fruits and vegetables with an association to antioxidants could prevent diseases and prolong life with vitamin A, β,β-carotene, vitamin E and vitamin C recognized [148–161]. Age-related diseases considered for antioxidants most vulnerable to ROS included cancers, cardiovascular disease, diabetes and neurological disorders [36,138,150,155,158,160,161].

Because of the numerous nutrition research trials done on diets high in vegetables and fruit demonstrating preventive improvements for disease, treatments for patients were regarded on the foundation of potential vitamin antioxidant actions to offset the damaging properties of ROS. Nevertheless, large vitamin clinical trials using supplements like vitamin A and β,β-carotene, vitamin E or vitamin C or several combinations have not demonstrated successful results in preventing cancer [161–169], cardiovascular disease [163,164,170–172] or diabetes [138,173]. In fact, to much concern a β,β-carotene cancer prevention clinical study that included 29,133 male smokers for an average of 6.1 years statistically significantly increased risk of lung cancer 18% and overall mortality [162]. The higher mortality rate was due to other pathology related to cardiovascular disease [162]. Another clinical trial that examined a combination of β,β-carotene and vitamin A with smokers and asbestos-exposed workers discovered a statistically significant 28% increase in lung cancer with the nutritional supplementation and 17% increase in total mortality that required the clinical study to conclude 21 months earlier than intended [164].

Despite the disappointing outcomes of the clinical trials with vitamin supplements, since diets high in fruits and vegetables with vitamin A and vitamin E lowered risks for cancer and cardiovascular disease, positive antioxidant properties may be related to nutrients other than vitamins not yet recognized [161]. Similar recommendations for diabetes emphasize the need for vitamins supplied from natural food sources by a balanced diet with alarm for potential harm from nutritional vitamin supplementation [138,173]. As an extremely important problem, antioxidant results for covalent bond crosslinking by polymerization shrinkage tests with vitamin A and β,β-carotene nutrition supplements both demonstrated exceedingly strong oxidative reactions by generating solids from low-viscosity oils when reacted with peroxide-derived free radicals [1] (Figure 4). Lowering analogous cell membrane fatty acid oil fluidity would reduce oxygen diffusion [66–68] and eventually create increased production of cellular free radicals during mitochondrial energy synthesis and related diseases with mitochondrial ROS pathology [2]. Immense inconsistencies between vitamin antioxidant potentials and clinical failures are due to the fundamental vitamin antioxidant tests using spectrophotometer methods [174–176]. Antioxidant spectrophotometer tests are based on optical color changes [174–176] that are the result of conjugated molecules adsorbing energy chiefly by bond stretching or bending and electrons moving to a higher-energy orbital [177] that is not an equivalent measure of biologic covalent bonding. Conversely, covalent bonds generated by reactive secondary sequence chemistry for oils as unsaturated oleic and linoleic fatty acid lipids (Figures 2 and 3), or vitamin A and β,β-carotene (Figure 4), that all produced rubbery solid gels or also a possible peroxidation crystalline lipid by molecular oxygen crosslinking (Figure 3) create a more accurate foundation for all major ROS pathology. In addition, free-radical reactive secondary sequence covalent chemistry or possible lipid peroxidation expose key failures in laboratory vitamin tests that inaccurately influenced the disappointing vitamin A and β,β-carotene clinical trials [1].

## Free-Radical Inhibitor Hydroquinone

Important antioxidant properties of fruits and vegetables may be derived from compounds other than vitamins not yet known [138,161]. The most familiar antioxidants for ROS are recognized as the enzyme cellular proteins superoxide dismutases, catalase and glutathione peroxidase [31,78,79,80] and can further include protein chains and peptide bonds that can delocalize radicals [2,3]. Also, coenzyme Q10/ubiquinone is a small molecule that carries electrons through the inner mitochondrial membrane in the electron transport chain which has been accepted as an antioxidant and so utilized as an over-the-counter nutritional supplement [178]. Additional quinones are developed for use in dermatology, food preservatives, and as antioxidants to safeguard chemicals in polymer manufacturing. Hydroquinone is utilized as a reducing agent, antioxidant, free-radical inhibitor for polymerization, food preservative and nonprescription skin lightener to treat hyper-pigmentation [179].

Hydroquinone epidemiological studies in a manufacturing plant with 9040 workers with an equivalent 94,524 survival years over about a 10-year period showed statistically significant decreases in mortality when evaluating exposed workers to both non-exposed plant workers and the common population [179,180]. The identical worker exposure study further showed statistically significant reductions in cancer rates, ischemic cardiovascular and cerebrovascular diseases, respiratory diseases and digestive diseases when evaluating state and national vital statistics [179,180]. An additional broad epidemiology study by 858 men with specific hydroquinone exposure for 22,895 person-years at another manufacturing plant for 48 years with an average contact of 13.7 years showed statistically significant decreases in mortality and cancer rates when evaluating both non-exposed plant workers and the common population [181,182]. More human exposure studies at a manufacturing plant with significant levels of hydroquinone dust contact demonstrated no systemic toxicity [179,183].

**Figure 8:**
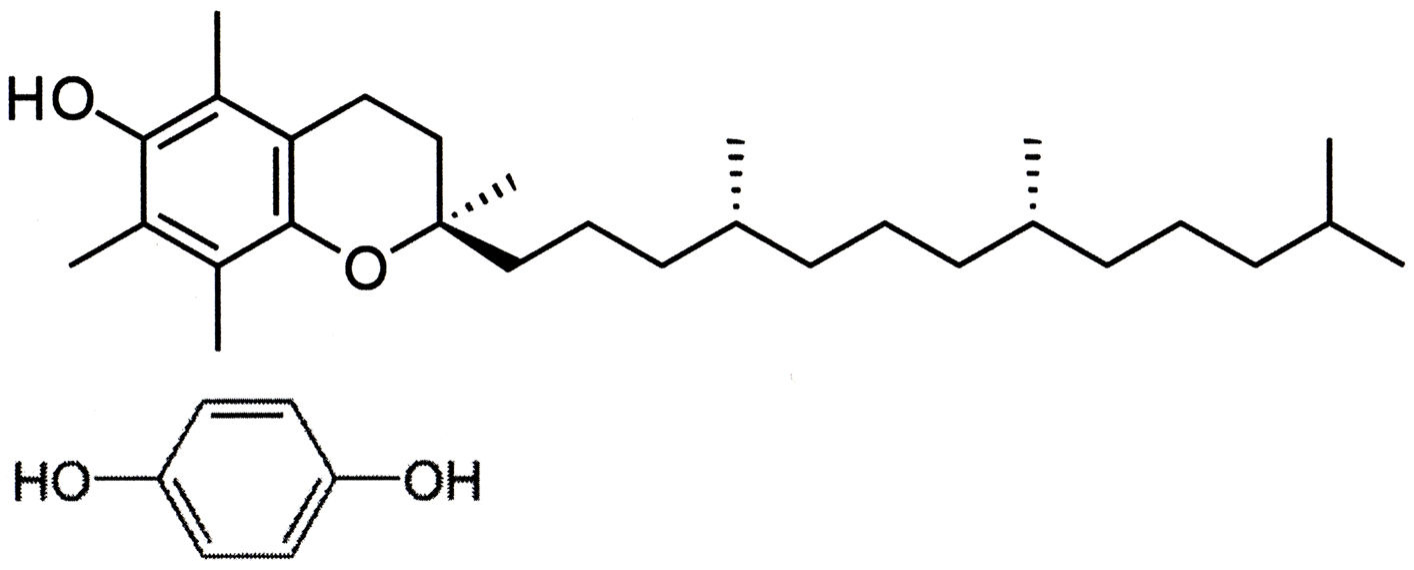
Molecular structures for vitamin E (top) compared to hydroquinone (bottom).

Vitamin E α-tocopherol compared to hydroquinone (Figure 8), has some molecular resemblance to hydroquinone with an aromatic hydroxyl group that greatly increases aromatic reactivity to possibly perform as an antioxidant. But, the aromatic ring for vitamin E is fully substituted with molecular groups. On the other hand, hydroquinone has four unsubstituted aromatic positions that can efficiently be activated toward ortho positions by two para substituent hydroxyl groups for reactivity with a strong electrophile as a free radical [184]. Two hydroquinone hydroxyl substituents must donate electrons by resonance stabilization to the aromatic ring so that electrons can flow from the oxygen lone pair electrons to add negative charges on the ring at the four possible open ortho positions for the hydroquinone molecule [184]. Consequently, electrophilic aromatic substitution reactions with hydroquinone or p-dihydroxybenzene would seem to be the main antioxidant chemistry to scavenge free-radical electrophiles [2]. Further, vitamin E α-tocopherol is a great deal larger hydrophobic or nonpolar molecule than hydroquinone and through laboratory observations is virtually insoluble in water while hydroquinone has solubility to easily diffuse through water [2].

**Figure 9:**
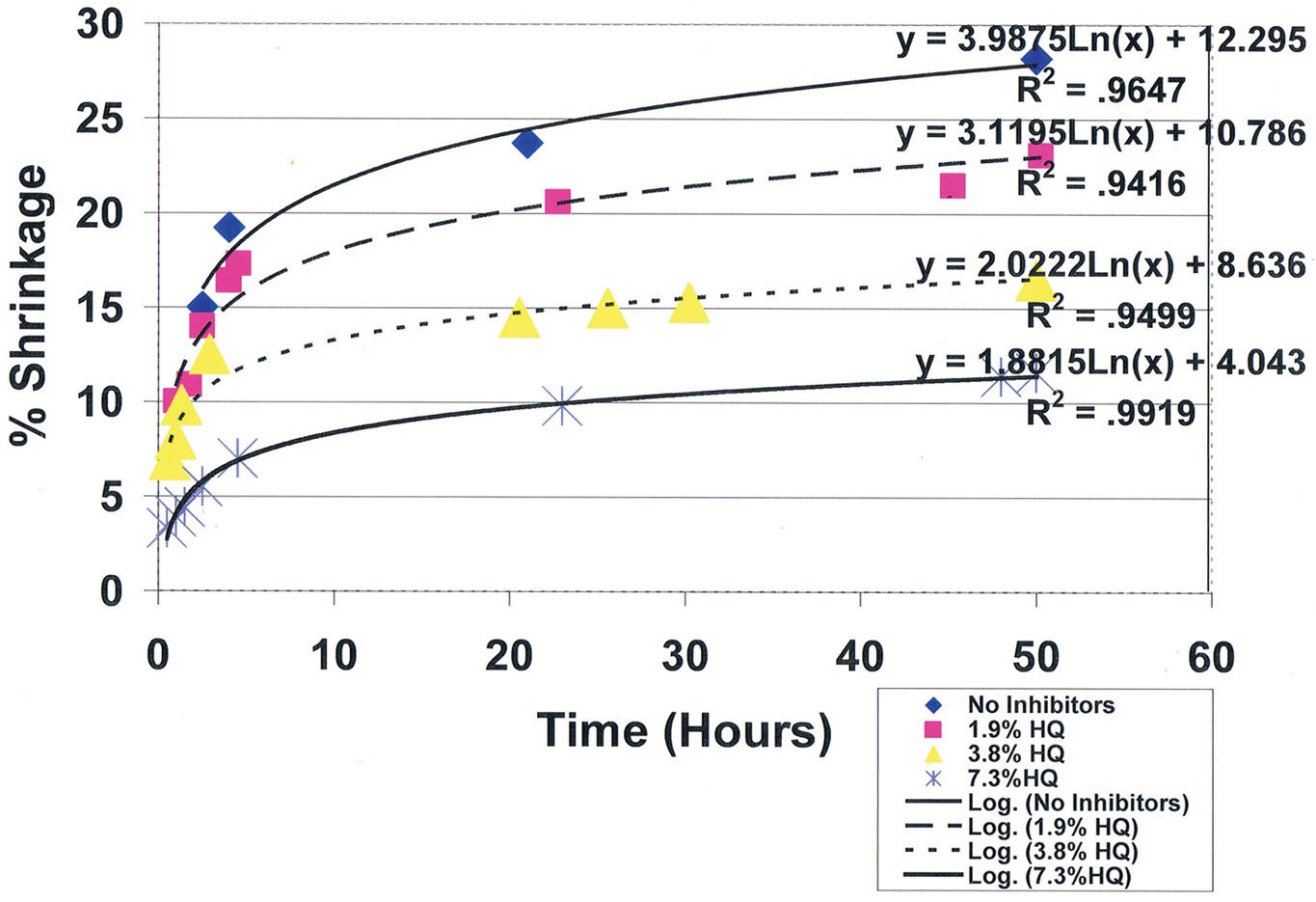
Unsaturated lipid and reactive acrolein free-radical covalent bonding polymerization shrinkage with hydroquinone free-radical inhibitor at different concentrations. (International Research Journal of Pure & Applied Chemistry 2(4): 247–285, 2012, Reactive Secondary Sequence Oxidative Pathology Polymer Model and Antioxidant Tests. Figure 15. Richard Petersen).

**Figure 10:**
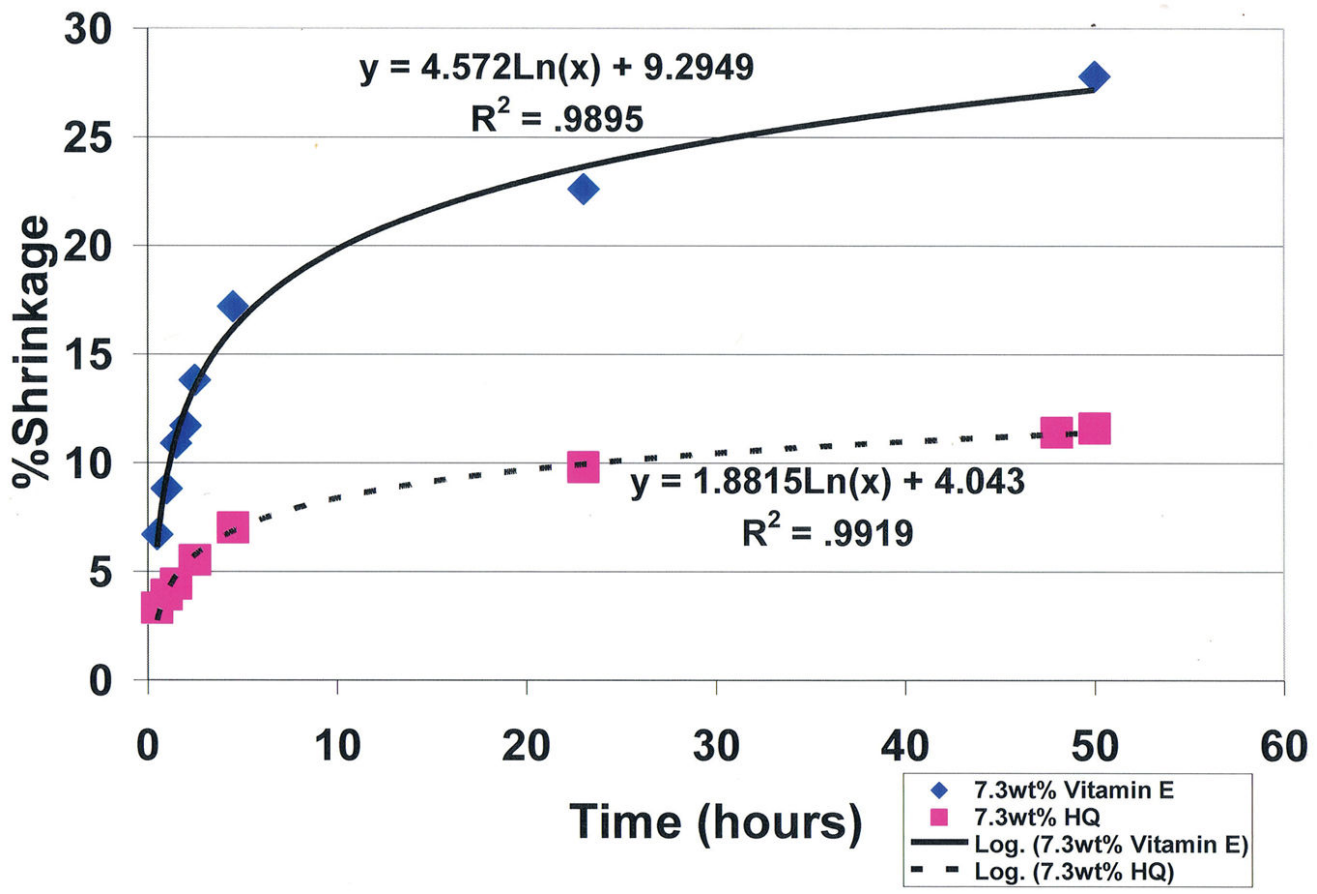
Unsaturated lipid and reactive acrolein free-radical covalent bonding polymerization shrinkage comparing antioxidant free-radical sequestering with 7.3wt% hydroquinone and 7.3wt% vitamin E. (*p*<0.00001 at 50hrs) (International Research Journal of Pure & Applied Chemistry 2(4): 247–285, 2012, Reactive Secondary Sequence Oxidative Pathology Polymer Model and Antioxidant Tests. Figure 16. Richard Petersen).

To compare the covalent-bond reaction for antioxidant properties between vitamin E and free-radical inhibitor hydroquinone, an unsaturated lipid and crosslinking unsaturated reactive aldehyde acrolein mixture were combined with the Fenton redox couples benzoyl peroxide initiator 4 wt% and cation transition metal cobalt naphthenate accelerator 4wt% to create free radicals [1]. For evaluations, equal control groups were combined with different weight percents of either vitamin E ((±)-α-tocopherol) or hydroquinone [1]. Polymerization shrinkage was then measured over a period of 50 hours by quantifying the differences between the original levels for the lipid reactant mixture volumes with the volumetric shrinkage polymerization levels as a comparative measure of covalent bond crosslinking. Results for hydroquinone demonstrated remarkable statistically significant increased antioxidant properties for removing free radicals with reductions in polymerization shrinkage during 50-hours of testing from the 28.2% control at 0.0wt% down to 11.6% at 7.3wt% (*p*<0.0001) (Figure 9). Antioxidant comparisons demonstrated an enormous statistical significant increase in free-radical inhibition for 7.3wt% hydroquinone over 7.3wt% vitamin E that showed virtually no antioxidant activity in scavenging free radicals by polymerization shrinkage measurements of 27.8% after 50 hours, (*p*<0.00001). Hydroquinone and vitamin E are compared simultaneously at 7.3wt% each in Figure 10. Nonetheless, vitamin E appears to have beneficial properties other than as an antioxidant, for example as a viscosity reducer [1,2].

## Conclusions

Free radicals generated under mitochondrial oxidative stress are associated with excess production of electrons and acid. Combined acid and free radicals with appropriate enzymes can break unsaturated lipids down into reactive unsaturated aldehydes of lower molecular weight. Subsequent reactive unsaturated aldehydes can then greatly help crosslink carbon-carbon double bonds by a reactive secondary sequence free-radical chain growth polymerization and even crosslink with O_2_ to form crystalline lipid peroxidation products. Crosslinking decreases the respective fluidity of lipids or even forms solid structure that reduces or blocks O_2_ diffusion. Subsequent lower O_2_ diffusion to mitochondria during energy synthesis increases more generation of free radicals and acids in possible continuing vicious cycles toward creating or maintaining most pathology known to mankind. Free radicals are also associated with crosslinking proteins and collagen to stiffen the extracellular matrix in much pathology. Most importantly, hydroquinone, a free-radical inhibitor designed to efficiently sequester free radicals, is a potential pharmaceutical for medical treatment.

## Abbreviations

O_2_: Molecular Oxygen
ROS: Reactive Oxygen Species
O_2_^•−^: Superoxide Anion
H_2_O_2_: Hydrogen Peroxide
HO^•^: Hydroxyl Radical
PUFAS: Polyunsaturated Fatty Acids
EMT: Epithelial-Mesenchymal Transition
